# Biomarkers Discovery for Colorectal Cancer: A Review on Tumor Endothelial Markers as Perspective Candidates

**DOI:** 10.1155/2016/4912405

**Published:** 2016-11-14

**Authors:** Łukasz Pietrzyk

**Affiliations:** ^1^Department of Didactics and Medical Simulation, Chair of Human Anatomy, Medical University of Lublin, Lublin, Poland; ^2^Department of General, Oncological and Minimally Invasive Surgery, 1st Military Clinical Hospital, Lublin, Poland

## Abstract

Colorectal cancer (CRC) is the third most common cancer in the world. The early detection of CRC, during the promotion/progression stages, is an enormous challenge for a successful outcome and remains a fundamental problem in clinical approach. Despite the continuous advancement in diagnostic and therapeutic methods, there is a need for discovery of sensitive and specific, noninvasive biomarkers. Tumor endothelial markers (TEMs) are associated with tumor-specific angiogenesis and are potentially useful to discriminate between tumor and normal endothelium. The most promising TEMs for oncogenic signaling in CRC appeared to be the TEM1, TEM5, TEM7, and TEM8. Overexpression of TEMs especially TEM1, TEM7, and TEM8 in colorectal tumor tissue compared to healthy tissue suggests their role in tumor blood vessels formation. Thus TEMs appear to be perspective candidates for early detection, monitoring, and treatment of CRC patients. This review provides an update on recent data on tumor endothelial markers and their possible use as biomarkers for screening, diagnosis, and therapy of colorectal cancer patients.

## 1. Introduction

Colorectal cancer (CRC) is the third most common cancer in the world with approximately 1.4 million new cases diagnosed in 2012. The disease is a leading cause of death in approximately 50% of CRC patients [1]. The predictions are that the morbidity and mortality rates of CRC will increase due to unprecedented global trends in population aging and profound adverse effects of many lifestyle-related factors [2]. Overall 5-year survival for cancer limited to the colon is 95% and 82% for stages I and stage II, respectively. However, it decreases considerably to 61% for patients with regional spread to the lymph nodes (stage III) and only 8% for patients with distant metastases (stage IV) [3]. The early detection of CRC, before the promotion/progression stages, is an enormous challenge for a successful outcome and remains a fundamental problem in clinical approach [4]. Despite the continuous advancement in diagnostic and therapeutic methods (i.e., colonoscopy, flexible sigmoidoscopy, and stool-based tests), effective in the reduction of mortality, the figures published by the National Cancer Institute indicate that still a significant number of individuals are diagnosed in later stages of CRC development (stages III and IV) [5].

Therefore, recent studies have been focusing on identification of sensitive and specific, noninvasive biomarkers that could detect the presence of CRC before it reaches advanced stages [6].

Recently a considerable attention is given to tumor endothelial cells (TECs). Abnormalities between tumor and normal endothelial cells open an opportunity to identify specific markers (tumor endothelial markers (TEMs)) linked to tumor angiogenesis. Markers that could distinguish physiological and pathological angiogenesis are an important issue for cancer detection [7, 8]. It is widely accepted that biomarkers offer chances to establish prognostic indicator in CRC and for their perspective use in clinical applications. Inhibiting angiogenesis is an important strategy for current therapies of cancer patients [9]. Selective delivery of blocking molecules to tumor endothelium has become a major goal of current antiangiogenic treatment strategies for cancer. An ideal marker for such selective targeting would be highly expressed in tumor endothelium but absent or exceedingly rare in all nontumor endothelium. To date, few, if any, markers have been identified that meet such strict criteria.

This review provides an update on recent data on tumor endothelial markers (TEMs) and their possible use as biomarkers for screening, diagnosis, and therapy of colorectal cancer patients.

## 2. Cancer Angiogenesis

There are two distinct stages of tumor development: (i) the avascular growth phase and (ii) the vascular growth phase. In the first phase, the tumor is solid (size < 1-2 mm, multicellular spheroids) and is dormant, noninvasive (carcinoma* in situ*). In the second phase, the spread of cancer may appear. Numerous studies have shown that avascular tumors are restricted in their growth potential due to the lack of a blood supply needed for cell-to-cell diffusion of nutrients and metabolites exchange. It is accepted that the solid tumor growth (size > 1-2 mm) and its invasion are angiogenesis-dependent [10].

The tumor may persist dormant for years after the primary neoplasm has been developed [11]. A significant turn-off point for the cancer growth, progression, and metastatic spread of cancer cells is angiogenesis [12]. The initiation of local small blood vessels development induces rapid, logarithmic tumor growth that follows tumor cells blood supply. The angiogenesis is a complex (multistage) process of new blood capillaries formation. The key stages include (i) initiation by endothelial cell activation (ECs), (ii) degradation of basement membrane, extracellular matrix, and pericyte detachment, (iii) proliferation and ingratiation of endothelial cells into the surrounding matrix, (iv) extensive enlargement of new vessels and stabilization of network (anastomosis), and (v) blood flow [12, 13].

The tumor blood vessels may have different origins. They may originate from preexisting neighboring blood vessels (termed “sprouting” angiogenesis) and from bone marrow-derived endothelial cell (EC) precursors (termed vasculogenesis) [14]. The mechanisms by which tumors promote new blood vessel formation are a complicated process that involves a complex and dynamic interaction between endothelial cells (ECs) and the corresponding extracellular and intracellular environment [15]. However, the molecular and cellular mechanisms that regulate angiogenesis have not been entirely explained. Many hypotheses have been proposed to clarify the tumor survival, the tumor growth, and its metastasizing to distant organs [12, 16]. For example, basement membrane degradation and pericytes detachment are provoked by upsetting of the local cathepsin-cysteine protease inhibitor balance [17]. Rapid enlargement of vessel's size is related to, at least in part, the transport of macromolecules across venules in the vascular matrix. The vesiculovacuolar organelles are involved in the process [18, 19]. The tumor blood vessels are dramatically different from normal blood vessels. These abnormalities involve morphological and functional disturbances is shown as follows [16, 20–24].

 Morphological and functional characteristics of tumor blood vessels are as follows.

*Morphology*

It has disorganized structure without branchial pattern of arteries, veins, and capillaries.
It does not form monolayer of endothelial cells.
It has abnormal basement membrane of loose association with endothelial cells and varying thicknesses of type IV collagen layers.
Pericytes with free association with endothelial cells have cytoplasmic processes extending deep into the tumor tissue.

*Function*

It might collapse and impedes blood flow.
It does not have normal barrier function.
It is characterized by increased leakiness.

The most widely accepted model for the tumor growth is an angiogenic model. It suggests the tumor “switch on” and the tumor growth dependent on the balance of endogenous proangiogenic and antiangiogenic factors [25]. The mechanisms that lead to angiogenic phenotype activation (tumor “switch on”) are associated with the expression of proangiogenic genes activated by production, secretion, and accumulation of proangiogenic factors that promote increased angiogenesis.

Therefore, one of the primary challenges for CRC early diagnosis is to identify a biomarker or a group of biomarkers that corresponds to early cancer stage of angiogenesis. Although angiogenic CRC-specific blood biomarkers have not been identified so far, several growth factors and their specific receptors have been recognized to induce and regulate angiogenesis in colorectal cancer. The most frequently described proangiogenic tumor “switch on” factors include vascular endothelial growth factors (VEGFs), fibroblast growth factor (FGF), platelet-derived growth factor (PDGF), transforming growth factors (TGFs), epidermal growth factor (EGF), angiopoietins (Angs), and others [6, 26]. Many* in vitro* models have shown that these molecules supported tumor angiogenesis and revealed that the degree of tumor angiogenesis is often closely linked to the level of these stimulating factors' concentration [11, 15, 17–19, 25, 26].

## 3. Tumor Endothelial Cells (TECs)

Histological abnormalities in blood vessels refer to the endothelium, pericytes, and basement membrane. Tumor endothelial cells (TECs) overexpress specific genes, such as tumor endothelial markers (TEMs) and epidermal growth factor receptors (EGFRs). The signaling system involves molecules that impact on pericyte/endothelial cell homeostasis [27, 28]. Abnormal expression patterns of biomolecules (proteins, glycoprotein, and glycans) are well known to be specific for tumor endothelium. The endothelial cells change their properties depending on their origin, age, and the expression of cell surface antigens. Circulating endothelial cells (CECs) are derived from multiple sources, including bone marrow (circulating endothelial progenitors (CEPs)) and established vasculature (mature CECs). As a result of respective complements of cell surface molecules and receptors, the functions of endothelial cells are diverse [17].

## 4. Endothelial Progenitor Cells (EPCs)

A growing body of evidence indicates the endothelial progenitor cells (EPCs) are particularly important for tumor angiogenesis determining tumor growth and metastasis [29, 30]. EPCs have been first described in detail by Asahara et al. [31]. EPCs hierarchy is defined based on their behavior, proliferative potential, and vasculogenic ability [32]. In human, main populations of EPCs include early EPCs and putative adult EPCs, circulating or resident cells. However, they can achieve at least 100 population doublings (PDs).

EPCs express a variety of cell surface markers similar to those expressed by vascular endothelial cells, adhere to endothelium at sites of hypoxia/ischemia, and participate in new vessels formation [33]. Early EPCs (localized predominately in the bone marrow) are positive for CD133 (termed AC133, human prominin-1 surface antigen initially), CD34 antigen, and VEGFR-2 (vascular endothelial growth factor receptor-2). More mature EPCs (circulating in blood) express phenotype CD133(−)/CD34(+)/VEGFR-2(+). Mature EPCs display a phenotype VEGFR-2(+)/VE-cadherin(+)/von Willebrand factor (+)(vWF) [34]. Furthermore, circulating in blood EPCs coexpress various molecules (platelet endothelial cell adhesion molecule-1 (PECAM termed as CD31)), CD146 immunoglobulin, and VE-cadherin (vascular endothelial) with different intensity [31, 34, 35]. These changes in antigens expression in endothelial progenitor cells suggest that at least two types of EPCs are present in the blood simultaneously and indicate that EPCs can change their properties in the blood [35]. It is speculated that EPCs potentials for proliferation and transformation into more mature EPCs are important in regulating the angiogenesis [36, 37]. However, it is still unclear at which point residual EPCs are induced to change into circulating EPCs, the endothelial-like subtype with angiogenic properties.

Furthermore, most of the endothelial markers used in assessing angiogenesis are not specific enough and are expressed in both normal and tumor tissues. However, a significant contribution to the issue has been made by Duda et al., who succeed in distinguishing between circulating endothelial cells (CECs) and leukocytes by using CD146 immunoglobulin in the blood of cancer patients [38].

## 5. Tumor Endothelial Markers (TEMs)

Due to the technical difficulties and problems with analyses of gene expression isolated from tumor endothelial cells, the studies on the tumor angiogenesis were carried out using normal endothelial cells (e.g., human umbilical vein endothelial cells) for a long time. The first who compared the gene expression in normal endothelial cells (ECs) and tumor endothelial cells (TECs) derivative from malignant colorectal tissues were St Croix et al. [39]. The serial analysis of gene expression (SAGE) in endothelial cells derived from blood vessels of normal cells and malignant colorectal tissues revealed 46 transcripts specifically elevated in TECs [40]. Nine transcripts were hoped to be unique to TECs and therefore were named tumor endothelial markers (TEMs). TEMs are potentially useful to discriminate between tumor and normal endothelial cells as 20-fold higher expression has been reported in human tumor endothelium [41]. It is speculated that tumor endothelial markers are likely to be most accessible to pharmacological agents, which is why TEMs are indicated as useful for pharmacological interventions in therapeutic targets [42].

Tumor endothelial markers (TEMs) belong to a family of proteins that are associated with tumor-specific angiogenesis [7, 8]. Genes encoding TEMs display elevated expression during tumor angiogenesis and are conserved in mice and humans. Therefore, TEMs are potentially useful to sufficiently distinguished patients with cancer and noncancer as well as cancer patients with different tumor stages. However, some studies pointed out that some TEMs might be even overexpressed during physiological angiogenesis; for example, TEM2 and TEM6 and their expression are not restricted to tumor endothelial cells [40].

**Figure 1:**
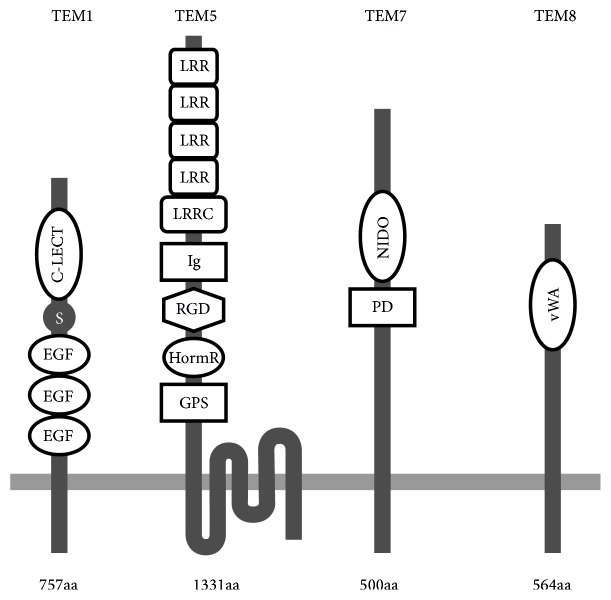
Predicted molecular structure of the tumor endothelial markers (TEM1, TEM5, TEM7, and TEM8). EGF: epidermal growth factor-like domain; S: Sushi domain; C-LECT: C-lectin domain; GPS: proteolysis site; HormR: hormone receptor domain; RGD: Arg-Gly-Asp motif; Ig: immunoglobulin-like domain; LRRC: leucine-rich repeat C-terminal domain; LRR: leucine-rich repeat; PD: plexin-like domain; NIDO: nidogen-like domain; vWA: von Willebrand type A domain; aa: amino acids chain length.

Studies investigating the expression of TEMs in human colorectal cancer (CRC) are limited. However, the most promising TEMs for oncogenic signaling in CRC appeared to be the TEM1, TEM5, TEM7, TEM7R, and TEM8. These tumor endothelial cells' markers display elevated expressions during tumor angiogenesis in CRC patients [8, 41, 43]. TEMs are transmembrane proteins containing putative domains that function as receptors; for example, TEM5 appears to be a seven-pass transmembrane receptor, whereas TEM1, TEM7, and TEM8 pass the cell membrane once (Figure 1) [44, 45].

### 5.1. Tumor Endothelial Marker 1

Tumor endothelial marker 1 (TEM1, endosialin, and CD 248) is a 165 kDa transmembrane glycoprotein of 757 amino acids. TEM1 consists of two domains: (i) a long-chain (670 amino acids) extracellular domain (ECD) and (ii) a short-chain (49 amino acids) cytoplasmic domain. The extracellular domain (ECD) of TEM1 consists of one C-type lectin-like, one Sushi, three EGF-like domain, and mucin-like stalk receptors. TEM1 has been localized on healthy and tumor endothelium and in stromal fibroblasts [43, 46]. The expression of TEM1 is specifically higher in tumor endothelium (5-, 10-, and 20-fold) compared to the endothelium of normal tissues [39]. A significant increase of TEM1 expression in the stroma between distant or adjacent normal mucosa and primary tumor (3%, 5%, and 63%, resp.) was documented in rectal cancer patients [47].

Two mechanisms are suggested to regulate TEM1 gene expression: (i) cell density and (ii) hypoxia [48, 49]. Opavsky et al. [48] observed the positive correlation of endogenous TEM1 expression with the density of NIH3T3 cells. Low level of TEM1 RNA was found in the sparse NIH3T3 cells. In contrast, the strongest induction of transcription was characteristic for NIH3T3 cells at full confluence. The upregulation of TEM1 expression is known to be induced by hypoxia, a condition well identified as a primary activator of angiogenesis in solid tumors [50]. Human cell lines (FIB-3 placental fibroblasts and 42-MG-BA glioblastoma cells) exposed to hypoxia (2% O_2_) were found to have significantly higher TEM1 gene expression compared to the same cell lines incubated in normoxia [49]. High TEM1 gene expression is partially mediated by an interaction between hypoxia-inducible factor-2 (HIF-2) and the Ets-1 transcription factor. Subsequent studies have confirmed that HIF-2 can activate the TEM1 distal (enhancer) and proximal (core) promoter. The combination of indirect and direct promoter activation by HIF-2-Ets-1 is suggested. Direct TEM1 distal promoter activation involves the HIF-2 binding to a hypoxia-response element (HRE) site and the adjacent ETS-1-binding site. The indirect pathway engages binding the Ets-1 and its two cognate EBS elements located in the proximal promoter of TEM1 [49, 51, 52].

TEM1 shows overexpression in tumor tissues. It was suggested that TEM1 is engaged in tumor invasion (progression and metastasis) all the more so because the absence of TEM1 expression reduced tumor growth [43]. Nanda et al. [53] using mouse xenograft model have shown an important functional role of TEM1 in growth and progression of abdominal tumors. The effect of Tem1 knockout (KO) on embryo development and vascularization in a wound healing assays (the wound sites morphology, number, and size of vessels at the site of the wounds) was not found. However, a considerable change in tumor growth pattern (alteration in vascularization, a decrease in bulk growth, prevention of the local invasion, and metastasis reduction) in HCT116 human colorectal cancer cells implanted into the serosal surface of the large intestines of Tem1 knockout (KO) mice has been documented. The lack of TEM1 gene expression resulted in the reduction of xenograft tumors aggressiveness; that is, smaller tumor volume and lower metastases rate (KO versus WT mice was 0% versus 33% of liver metastases) have been revealed [53].

TEM1 is elevated in a wide range of human carcinomas (breast, lung, pancreas, urinary bladder, brain glioma, and melanoma) [43, 54–58]. Rmali et al. [43] found overexpression of TEM1 in colon cancer tissues compared to normal tissues (95.5% versus 38% of positive tissues for TEM1 expression, resp.; *p* < 0.01). Zhang et al. [47] reported significantly lower expression of TEM1 in rectal cancer tissues when comparing TNM stage I with other stages: TNM II + III + IV (45% versus 74%, resp.; *p* = 0.03). Accordingly, it was suggested that, in CRC patients, TEM1 expression correlates with the disease' advancement [43].

The elevated expression of TEM1 has also been found in pericytes (vascular cells that envelop the surface of the vascular tube) [54, 59]. Pericytes are considered to be directly involved in regulation of blood vessels morphogenesis and play a critical role in cardiovascular homeostasis [28, 60]. Evidently, modulation of pericyte function is likely to reduce normal and pathological angiogenesis. The absence of TEM1 expression in pericytes causes a decrease of larger and mature vessels and an increase of small and immature tumor vessels, indicating a considerable involvement of TEM1 in tumor angiogenesis and microvasculature maturation [53, 61]. Tomkowicz et al. described the molecular mechanism by which the tumor-stromal compartment can control tumor aggressiveness [54]. The suggested mechanism includes three phases: (i) an activation of TEM1 expression in perivascular cells and stromal fibroblasts by unknown factor, (ii) a modulation of tissue proteases resulting from interaction between TEM1 and extracellular matrix components that is pericytes or fibroblasts, and (iii) tumor vessels maturation and induction of cell-to-cell attachments and cells migration.

Since the expression of TEM1 in colorectal carcinogenesis has been documented, the TEM1 has become a potential diagnostic and therapeutic target molecule [39]. Preclinical murine model of ID8 tumors (a mouse ovarian surface epithelium cancer cell line) designed by Chacko et al. [62] showed the potential diagnostic utility of MORAb-004, a humanized IgG1/*κ* monoclonal antibody (mAb) directed against human TEM1. In the immuno-PET examination, high specific and sensitive uptake of 125I-MORAb-004 and 124I-MORAb-004 in tumors compared to healthy tissue was observed (immunoreactivity approximately 90%). Moreover, high tumor-to-background tissue contrast was achieved for 124I-MORAb-004. Similar immunoreactivity was demonstrated with 89Zr-MORAb-004 [63]. 89Zr-MORAb-004 was able to bind TEM1 and could distinguish between high and low TEM1 expression tissues, which has been evidenced in specific immuno-PET imaging of sarcoma cell lines xenografts [63].

St Croix et al. also pointed out the TEM1 was a promising prospect for use in cancer immunotherapy [39]. They showed that TEM1 was recognized by the monoclonal antibody called FB5, detectable immunohistochemically on blood vessels of a majority of human tumors. Moreover, Facciponte et al. [64] in murine colon cancer model (colon carcinoma line CT26) revealed significant tumor protection effect of TEM1-TT vaccine (TEM1 cDNA fused to the minimal domain of the C fragment of tetanus toxoid). The TEM1-TT vaccine showed the power to break down TEM1 tolerance. TEM1-TT vaccination exerted humoral antitumor activity through the significant increase of CD3+ T cells infiltration in the tumor (42 ± 5 CD3+ cells/high-powered field [hpf] versus TT-vaccinated mice; *p* < 0.05). TEM1-TT-vaccinated tumors were characterized by significantly lower hemoglobin level and showed decreased area of perfusion and blood flux compared to TT-vaccinated tumors. The dependence suggests the functional disruption of tumor vasculature induced by TEM1-TT vaccine. Currently, clinical studies (phase I) are conducted to assess both biological and side effects of the MORAb-004 in patients with solid tumors (*n* = 36), including colorectal cancer patients (*n* = 11) [65]. The MORAb-004 maximum tolerated dose at the level of 12 mg/kg has been defined, and treatment-emergence adverse events (i.e., fatigue 47.2%, headache 36.1%, pyrexia 22.2%, chills 19.4%, and nausea 13.9%) have been noticed. Antitumor activity with stabilization of the disease at least for 106 days in patients with tumor of epithelial origin, including colorectal cancer patients, has been documented [65]. The mechanism of MORAb-004 action is not thoroughly revealed. However, the MORAb-004 mediated internalization and removing of TEM1 from the cells surface have been suggested as a possible one. The TEM1 involvement in the interaction with extracellular matrix proteins (i.e., fibronectin (FN), collagen types I and IV) and/or participation in signaling pathways (i.e., via the PDGF-receptor) are considered. Presumably, MORAb-004 controls TEM1 adhesion to the extracellular matrix or regulates the TEM1 involvement in cell signaling pathways. In these ways, the MORAb-004 indirectly influences the tumor-stromal cells communication. The phase II clinical studies have been designed and initiated in patients with colorectal cancer, melanoma, and soft tissue sarcoma to answer a role of MORAb-004 in adhesion, migration, survival, and proliferation of tumor [54, 65–67].

The TEM1 expression seems to be a promising target for cancer diagnosis and immunotherapy. The TEM1 expression could be detected by MORAb-004 in positron emission tomography. This allows classification and selections of patients with high TEM1 expression for more detailed diagnosis. Regarding that the MORAb-004 can inhibit endosialin/TEM1-mediated interaction with extracellular matrix, the MORAb-004-based immunotherapeutic product may block TEM1 and reduce carcinogenesis [63, 65].

### 5.2. Tumor Endothelial Marker 5

Tumor endothelial marker 5 (TEM5, G-protein-coupled receptor 124, and GPR124) is homologous to adhesion G-protein-coupled receptors (GPCRs) and belongs to a group of integral transmembrane proteins. TEM5 is composed of 1331 amino acids [68]. The protein bears long extracellular N-terminal part (N60), seven-pass domains, and an intracellular domain the C-terminal part (C50). TEM5 extracellular domain [termed sTEM5 (soluble TEM5)] is composed of subdomains and motifs, that is, an immunoglobulin (Ig) domain, a hormone receptor (HormR), cryptic RGD (Arg-Gly-Asp) motif, a leucine-rich repeat (LRR) domain, a leucine-rich repeat C-terminal (LRRCT) domain, and a membrane proximal GPCR proteolysis site [69].

TEM5 activation is related to the cell-to-cell contacts during capillary morphogenesis [28]. TEM5 subdomains and motifs are involved in cell-cell and cell-matrix interactions. For example, RGD motif by binding the specific integrin (i.e., *α*v*β*_3_) receptors on the surface of endothelial cells modulates cell membrane functions, for example, adhesion, migration, and mediates endothelial cell survival during angiogenesis [70]. The C-terminal part (C50) domain interacts with the tumor suppressor protein human disc large (hDlg) [69]. It has been reported that the hDlg protein is involved in cell growth control and is known as a negative regulator of cell proliferation [71].

Interestingly, during capillary formation, TEM5 expression and upregulation can be induced by GTPase/Rac cascade [68]. It is currently clear that the cell proliferation in endothelial human umbilical vein endothelial cells (HUVEC) can be inhibited by blocking monoclonal TEM5 antibody. This creates the potential for the inhibition of cell divisions in endothelial tissues during capillary morphogenesis. Another study demonstrated a vital role of TEM5 in coactivation of the signaling pathway in endothelium brain development [72]. This fact together with TEM5 overexpression in tumor vasculature holds promise for the creation of new drugs that will block angiogenesis and cancer growth [41].

TEM5 architecture partly helps to explain multiple mechanisms of its action. The protein binds ligands (i.e., secretin, calcitonin) and activates many specific receptors located on the cell surface or inside the cell. In turn, TEM5 receptors are engaged in signal-transduction cascades and have the capacity for generation of mitogenic signals via currently only partly understood pathways. TEM5 is involved in the regulation of adenylate cyclases (AC) signaling system [68]. Consistent with this observation, TEM5 role in cell signaling pathways occurring during multiple cancer types development and progression is proposed. One of the presumable TEM5 actions is assistance in the interaction between the linked receptors and tyrosine kinase receptors, resulting in kinase inhibition [69]. Kinases overexpression enabled CRC progression and shortened cancer-specific survival [73]. These findings suggest that TEM5 is the important regulator in CRC spread [71].

TEM5 was highly expressed in endothelial cells and tumor stroma in human colon carcinoma xenograft compared to normal colonic tissue [41]. The expression of TEM5 is found during capillary morphogenesis or capillary network formation [27, 28]. However, evidence for the TEM5 role in tumor angiogenesis has not been explained. Current report suggests TEM5 plays a key role in VEGF-induced tumor angiogenesis. Mouse xenograft tumor angiogenic vessels formation and in turn tumor growth were inhibited by silencing TEM5 in human endothelial cells. TEM5 regulated VEGF-induced tumor angiogenic processes* in vitro* including cell-cell interaction, permeability, migration, invasion, and tube formation [74].* In vitro* study showed that knockdown of TEM5 in non-small cell lung cancer (NSCLC) reduces resistance to gefitinib [75]. This result indicates that TEM5 may contribute to the pathogenic angiogenesis. Hence, it appears to be a potential therapeutic target.

### 5.3. Tumor Endothelial Marker 7

Tumor endothelial marker 7 (TEM7, plexin domain containing 1, and PLXDC1) belongs to a group of transmembrane proteins. The protein contains 500 amino acids that form a large extracellular nidogen-like domain, a hydrophobic transmembrane domain, and a short cytoplasmic tail [76].

The increased TEM7 expression was established in aortic endothelial cells in mouse models [77]. Further, these authors have proven the TEM7 role in the capillary morphogenesis. Based on the reverse transcriptase-polymerase chain reaction (RT-PCR), TEM7 importance in the formation of functional microvascular proliferation and maintenance of neovascular endothelial cells in the fibrovascular membranes (FVMs) was shown [78]. These results suggest the crucial role of TEM7 expression in tumor-related blood capillaries formation. The modulation of TEM7 expression seems to be essential for blocking tumor capillaries to inhibit tumor growth [77].

TEM7 was first identified by St Croix et al. who found specific overexpression of this molecule in the endothelium of colorectal cancer [39]. Similarly, Rmali et al. [43] found the significantly higher expression of TEM7 in the tissue of colorectal cancer compared to healthy tissue (77.5% versus 15% of positive tissue with TEM1 expression, resp.; *p* < 0.04). Consistent with these observations, significantly higher detection of TEM7 transcripts in colon carcinoma tissues than in the normal colon tissue (85% versus 21% of TEM7 ISH+, resp.) was identified using* in situ* hybridization techniques performed on frozen tissues [79]. Considering these results, the TEM7 is suggested to be an attractive and perspective prognostic marker in CRC patients. The number of TEM7 transcripts was described to be higher in Dukes C compared to Dukes A colorectal tumor [43]. In this study, high level of TEM7 transcripts was associated with lymph nodes involvement and cancer progression. Knockdown of TEM7 resulted in a reduction of cell migration and invasion [80]. In gastric cell lines, downregulation of TEM7 by siRNA showed significant inhibition of gastric cell migration, approximately 50% in SGC-7901 cell line and 60% in AGS cell line. Moreover, TEM7 knockdown resulted in decreased cells invasion by 70%, which led to reduced metastasis.

Except for overexpression of TEM7 in colorectal cancer, tests revealed high upregulation of TEM7 in the endothelium and perivascular cells of primary human cancers of the lung, pancreas, breast, brain, and osteogenic sarcoma [8, 76, 81]. Besides, soluble and secreted forms of TEM7 have been identified [76]. After that, it has been found that TEM7 via extracellular nidogen-like domain can interact with cortactin cytoplasmatic protein, and the binding region is minuscule (nine-amino-acid sequence). TEM7-cortactin interaction is supposed to be useful in the development of low weight molecules (peptides or analogs) that can be a promising tool for diagnosis and treatment of cancer by targeting tumor endothelium [76, 82].

Targeting angiogenesis is recognized as an effective way to promote cancer cell death in cancer patients' treatments. Therapy with anti-TEM7 seems to be a novel approach. Antibodies against TEM7 might interfere and block exclusively tumor blood vessels with elevated expression of TEM7. In comparison to therapies targeting, that is, VEGF pathway, the anti-TEM7 therapy might result in a reduction of side effects of antiangiogenic therapies, that is, hypertension, cardiotoxicity, or proteinuria [83]. TEM7 targeted in human SKOV3 ovarian carcinoma and MDA-MB-231 breast carcinoma cells showed perspective effects [79]. An anti-TEM7 antibody caused lysis of the cancer cells. Percentage of lysis in anti-TEM7 targeted MDA-MB-231 cancer cells was higher than in SKOV3 cells (control assay) incubated with trastuzumab (74.8 ± 1.0% in MDA-MB-231 versus 61.8 ± 7.2% in SKOV3). These results provide a new insight for antiangiogenic therapy of patients with some cancers.

Despite the* in vivo* experiments, the therapy targeting TEM7 calls for the clinical trials that could prove the immunomodulatory activity of anti-TEM7 antibodies.

### 5.4. Tumor Endothelial Marker 8

Tumor endothelial marker 8 (TEM8, anthrax toxin receptor (ATR), and ANTXR1) is a type I transmembrane glycoprotein composed of 564 amino acids. TEM8 is homologous to capillary morphogenesis gene-2 (CMG2, ANTXR2), also a type I transmembrane protein. TEM8 and CMG2 amino acids sequence is similar in 40% [84]. Both TEM8 and CMG2 proteins contain the extracellular domain, the von Willebrand factor A (vWF). The vWF domain homology is 60% between TEM8 and CMG2. Within the vWF domain of the TEM8/CMG2 proteins a conserved metal-ion-dependent adhesion site (MIDAS) motif is located. Two MIDAS conformations are recognized: closed (low-affinity ligand binding state) and open (high-affinity ligand binding state) [85, 86]. A metal ion-dependent adhesion site (MIDAS) is critical for binding the receptors. The vWF domain performs its hemostatic functions through the interaction with transmembrane receptors (from integrins superfamily) that make the bridges for cell-to-cell and cell-to-extracellular matrix communications. Therefore, it has been identified as a unique endothelial marker in angiogenesis [87]. The interaction between TEM8 and cellular matrix components suggests a potential role of TEM8 in adhesion and migration of cells [88]. Loss of TEM8 expression in mice resulted in embryonic and postnatal vascular and connective tissue defects, which leads to extensive hemorrhage and has had an increasing effect on deposition of extracellular matrix [89]. Therefore, the TEM8 involvement in the regulation of the balance between proliferative and fibrotic processes that occur during angiogenesis is proposed.

However, TEM8 and CMG2 bind proteins (collagen type I, collagen type IV, and laminin) to extracellular matrix are different [78]. The TEM8 receptor is responsible for entry of anthrax toxin (produced by gram-positive bacterium* Bacillus anthracis*) into host cells and appears to regulate endothelial cell migration and tubule formation [85]. The CMG2 is involved in the homeostasis of the extracellular matrix [84]. These biochemical properties and the biologic behavior of TEM8 and CMG2 may be responsible for their apparent roles in regulating endothelial cell behavior during angiogenesis.

The presence of TEM8 on the cell surface is selectively controlled [87]. TEM8 is considered as a novel extracellular tumor marker among the other cell surface TEMs. TEM8 expression pattern is tumor-specific and has not been detected in physiologic angiogenesis [90, 91]. The TEM8 expression was found in various cancers, that is, breast and gallbladder [91–93]. Moreover, high TEM8 levels were associated with significantly shorter survival in breast cancer patients [91].

TEM8 is upregulated in tumor endothelium derived from human and mice colorectal tissue [41, 53, 94]. Overexpression of TEM8 was found in colon cancer tissue compared to normal colon tissue [95]. Additionally, TEM8 antibody showed the high TEM8 expression in microvessels of tumor tissue. Therefore, the TEM8 is recognized as a useful marker for identifying tumor-associated microvessels in CRC [95, 96]. Results of the study of Raeisossadati et al. strongly confirmed the usefulness of TEM8 as a biomarker for the detection of CRC patients [97]. Authors found significantly higher circulating mRNA levels of TEM8 in the peripheral blood of CRC patients (22/40) compared to healthy controls (9/40). It seems that TEM8 appears to be not only a promising marker of colorectal tumor presence but also the marker of tumor invasiveness and spread. Rmali et al. revealed an increased number of TEM8 transcript copies in colon cancer tissue in an advanced stage of disease (Dukes C: 73 ± 0.5 TEM8 transcript copies versus Dukes A: 32 ± 0.5 TEM8 transcript copies *p* = 0.01) [95].

Since TEM8 expression is specific for tumor vasculature, antagonists of TEM8 might disrupt tumor angiogenesis and inhibit tumor progression [39, 98]. Quan et al. [99] developed antibody (a 13-meric peptide, KYNDRLPLYISNP; QQM) able to bind with TEM8. The QQM peptide labeled with 18F bound specifically to the extracellular domain of TEM8 in both the head-and-neck cancer and melanoma models. This property could serve as a new target in cancer imaging and therapy [99, 100]. Chaudhary et al. [101] tested the L2 and L5, full IgG antibodies against TEM8 which was expressed in tumor xenografts including colon cancer (DLD1) in mice. The block of TEM8 expression resulted in tumor growth inhibition, similar to tumor growth impairment observed in the genetic ablation of TEM8. Fernando and Fletcher [94] evidenced that antibodies targeting TEM8 reduce tumor volume approximately of 53% and confirmed a tumor growth delay of 49% in the xenograft model of colorectal carcinoma compared to control mice. All of these data support the hypothesis that anti-TEM8 antibodies display potent antitumor activity. These antibodies are functionally involved in selective inhibition of angiogenesis and by that means indirectly block the tumor development [102].

The other molecules recognized as tumor endothelial markers and partially described are TEM2 and TEM4.

### 5.5. Tumor Endothelial Marker 2

Tumor endothelial marker 2 (TEM2, RASD family member 2): the protein functions as an activator of rapamycin 1 complex (mTOR1), which in turn is involved in multiple cellular functions, that is, regulating nutrient/energy/redox flow. Therefore, the activity of RASD/mTOR1 pathway controls the cell growth and proliferation by ensuring the resources and energy for protein synthesis [103]. The usefulness of TEM2 as an indicator for assessing the degree of tumor angiogenesis in colorectal cancer is limited. Although high expression of TEM2 is characteristic for colorectal cancer, it is also detected in the normal tissues (expression of TEM2: 45% in normal colon tissues versus 58.3% in tumor tissue; *p* > 0.05). However, significantly high transcript copies of TEM2 were found in Dukes A compared to Dukes C colorectal cancer [43, 55]. These patterns of TEM2 expression in cancer and healthy tissues reveal that TEM2 is not specific for tumor angiogenesis and makes the TEM2 doubtful marker in detection and targeting in tumor-related angiogenesis. Further investigations are needed to explore the TEM2 function in different tumors and to determine the possible role of TEM2 in tumor blood vessels formation.

### 5.6. Tumor Endothelial Marker 4

Tumor endothelial marker 4 (TEM4, ARHGEF17) is a Rho-specific guanine nucleotide exchange factor. It contains the DH and PH domains, extended N-terminal sequences with no identifiable domains or motifs, and a C-terminal domain [104]. TEM4 is localized in the subcellular compartment. The biological role of TEM4 is associated with regulating activation of Rho GTPases, various members of the cadherin-catenin complex, and several cytoskeleton proteins [105, 106]. TEM4 upregulation was identified in the endothelial cells during tumor angiogenesis of colorectal cancer patients [39]. Consistent with the TEM4 central role in controlling activation of RhoC in endothelial cells, the organization of actin cytoskeleton, cell-substrate adhesion, and cellular migration, it is suggested that TEM4 is critical for blood vessel formation during angiogenesis [105].

## 6. Conclusion

The correlation between predominant TEMs expressions in the endothelium strongly suggests a significant involvement of TEMs in tumor blood vessels formation. The development of biomarkers-based diagnostic tests is still a great challenge in cancer diagnosis and therapy. Overexpression of TEM1, TEM7, and TEM8 in colorectal tumor tissue compared to healthy tissue suggests their role in pathological angiogenesis. TEMs specifically expressed in tumor tissue provide potential novel targets to develop diagnostic and therapeutic molecules. Anti-TEMs antibodies provide a promising new tool for selective identification of pathological vessels. It appears that block of TEMs expression is a crucial therapeutic pathway in angiogenesis-dependent diseases, including colorectal cancer.

However, many questions about TEMs remain undiscovered and will need to be addressed in future molecular and clinical studies. Further dissection of the TEMs pathway should provide much insight into the molecular mechanism of cancer angiogenesis and its regulation.
